# Absolute substrate oxidation rates are lower in older adults with amnestic mild cognitive impairment

**DOI:** 10.14814/phy2.70326

**Published:** 2025-04-13

**Authors:** Nicholas A. Rizzi, Mary K. Kramer, Theodore M. DeConne, James M. Ellison, Alyssa M. Lanzi, Matthew L. Overstreet, David G. Edwards, Matthew L. Cohen, Curtis L. Johnson, Christopher R. Martens

**Affiliations:** ^1^ Department of Kinesiology and Applied Physiology University of Delaware Newark Delaware USA; ^2^ Department of Biomedical Engineering University of Delaware Newark Delaware USA; ^3^ Department of Psychiatry and Human Behavior Jefferson Health Philadelphia Pennsylvania USA; ^4^ Department of Communication Sciences and Disorders University of Delaware Newark Delaware USA; ^5^ Delaware Center for Cognitive Aging Research University of Delaware Newark Delaware USA

**Keywords:** amnestic mild cognitive impairment, exercise, substrate oxidation

## Abstract

Previous studies in individuals with mild cognitive impairment suggest that they may have altered systemic metabolic function at rest; however, metabolic function during aerobic exercise is not fully understood in this population. This study sought to determine whether individuals with amnestic mild cognitive impairment (aMCI) have lower rates of baseline and peak fat oxidation (FatOx) during a graded exercise test (GXT) compared with cognitively unimpaired control participants (CU). Twenty‐two (22) older adults with aMCI and 21 age‐ and sex‐matched adults completed a GXT to assess rates of substrate oxidation and peak oxygen consumption (VO_2_ peak). Rates of FatOx and carbohydrate oxidation (CHOOx) were assessed using VO_2_ and VCO_2_. Resting absolute (0.10 ± 0.03 vs. 0.09 ± 0.02 g/min, *p* = 0.126) and relative (1.5 ± 0.43 vs. 1.4 ± 0.44 mg/kg/min, *p* = 0.492) rates of FatOx, as well as resting absolute (0.51 ± 0.11 vs. 0.59 ± 0.15 g/min, *p* = 0.093) and relative (8.0 ± 2.3 vs. 7.5 ± 2.7 mg/kg/min, *p* = 0.126) rates of CHOOx were similar between groups. However, peak absolute rates of FatOx (0.33 ± 0.13 vs. 0.39 ± 0.10 g/min, *p* = 0.033) and CHOOx (1.9 ± 0.41 vs. 2.2 ± 0.49 g/min, *p* = 0.046) were significantly lower in the aMCI group. Time to fatigue (7.2 ± 2.0 vs. 8.7 ± 2.3 min, *p* = 0.033) and absolute VO_2_ peak (1.3 ± 0.34 vs. 1.6 ± 0.47 L/min, *p* = 0.024) were also significantly lower in the aMCI group. These findings suggest that absolute peak rates of whole‐body FatOx and CHOOx are reduced during aerobic exercise in older adults with aMCI.

## 1 INTRODUCTION

Mild cognitive impairment (MCI) is a condition in which cognitive functioning declines beyond what is expected for a person's age but does not significantly interfere with functional independence (*Alzheimer's & Dementia: The Journal of the Alzheimer's Association*, 2024; Petersen, 2004). Individuals with MCI may be further classified as having an amnestic or non‐amnestic subtype depending on whether memory functioning is abnormal (Petersen, 2007). Those with amnestic MCI (aMCI) are more likely to have underlying Alzheimer's disease (AD) pathology (Petersen, 2007). Many, but not all, individuals with MCI progress to having dementia later in life (*Alzheimer's & Dementia: The Journal of the Alzheimer's Association*, 2024). However, progression occurs at different rates (*Alzheimer's & Dementia: The Journal of the Alzheimer's Association*, 2024), and it is believed that secondary prevention efforts may still be effective at extending functional independence in individuals with MCI.

Consistent physical activity and exercise throughout the lifespan appear to have protective effects on cognitive functioning later in life (Hörder et al., 2018; Livingston et al., 2020; Singh‐Manoux et al., 2019). Such benefits are likely due, at least in part, to positive influences on risk factors like hypertension, obesity, and diabetes (Livingston et al., 2020). However, physical activity and exercise may also protect against future cognitive decline due to their positive influences on metabolic health (Memme et al., 2021). Recently, San‐Millán and Brooks assessed substrate oxidation rates during exercise in individuals with metabolic syndrome (San‐Millán & Brooks, 2018), a cluster of risk factors associated with an increased risk of both developing AD (Foret et al., 2020; Rojas‐Gutierrez et al., 2017) and progressing from MCI to dementia (Pal et al., 2018). They reported that individuals with metabolic syndrome have reduced rates of both submaximal and maximal fat oxidation (FatOx) compared to moderately active adults (San‐Millán & Brooks, 2018). Further, a shift away from FatOx and towards carbohydrate oxidation (CHOOx) occurred nearly immediately following the onset of exercise in those with metabolic syndrome (San‐Millán & Brooks, 2018). Considering that peak FatOx typically occurs between 45% to 65% of VO_2_ peak (Achten & Jeukendrup, 2004), this suggests that individuals with underlying metabolic dysfunction may be unable to increase FatOx to meet energy demands during even mild increases in energy expenditure, such as those that may occur during activities of daily living. Although the study lacked a true control group of sedentary adults (San‐Millán & Brooks, 2018), it supports the idea that underlying differences in metabolic function may be detected by examining substrate oxidation rates during exercise. Therefore, our study aimed to determine whether individuals with aMCI would display altered levels of FatOx at rest and during graded exercise compared to cognitively unimpaired older adults. We hypothesized that individuals with aMCI would display similar rates of baseline and peak CHOOx, but reduced rates of baseline and peak FatOx, compared to age‐ and sex‐matched cognitively unimpaired control participants (CU).

## 2 MATERIALS AND METHODS

### 2.1 Participants and aMCI classification

The data used in this study were collected as part of a larger clinical trial at the University of Delaware (NCT03855475). The parent study was approved by the University of Delaware's Institutional Review Board, and all participants provided written informed consent prior to participation. All participants were recruited from Newark, DE and the surrounding community. Prospective participants were recruited and screened for aMCI through the Delaware Center for Cognitive Aging Research. Briefly, targeted advertisements were placed by mail and online seeking older adults with concerns about memory loss. After screening for basic eligibility criteria, interested participants completed the modified *Telephone Interview for Cognitive Status* (TICS‐m) (Cook et al., 2009) as an initial screen for cognitive impairment. Individuals who scored between 21 and 34 points (out of the maximum possible score of 51) on the TICS‐m, or who produced a score ≤10 on the delayed recall component of the test, were considered most likely to have aMCI based on previous research on the sensitivity and specificity of the measure (Cook et al., 2009; Seo et al., 2011), and were therefore invited to complete additional, in‐person testing with the *Mini‐Mental State Exam, Second Ed*. (MMSE‐2) (Folstein et al., 2010), *Hopkins Verbal Learning Test‐Revised* (HVLT‐R) (Benedict et al., 1998), and the Logical Memory subtest from the *Wechsler Memory Scale, Fourth Ed*. (WMS‐IV) (Wechsler, 2009). Additionally, prospective aMCI participants who completed follow‐up testing were asked to select a study partner. Each prospective subject's study partner completed an interview to inform *Clinical Dementia Rating* (CDR) scores (Morris, 1993) to confirm a CDR Global score of 0.0 or 0.5. Upon completion of the follow‐up screening visit, MCI status was determined based on National Institute on Aging and Alzheimer's Association criteria (Albert et al., 2011). *Amnestic* MCI status was based on Petersen's criteria (Petersen, 2004), which required a memory score to be ≤−1.5 SD of an age‐matched normative group.

Individuals were excluded if they had a history of a major psychiatric disorder (e.g. schizophrenia, bipolar disorder), neurological or autoimmune conditions affecting cognition (e.g. Parkinson's disease, multiple sclerosis), other systemic illnesses (e.g. cardiovascular disease, cancer, renal failure), hypertension (systolic blood pressure >130 mmHg or diastolic blood pressure > 80 mmHg), had a recent history of smoking (within the past 3 months), or had a concussion within the last 2 years or ≥3 lifetime concussions. Additionally, because the primary endpoint for the parent clinical trial involved neuroimaging, participants were excluded from this study if they were unwilling or unable to complete MRI scanning. Habitual levels of physical activity prior to enrollment were not assessed as part of the inclusion criteria. For the present study, we analyzed baseline exercise testing data from 22 older adults with aMCI and 21 age‐ and sex‐matched cognitively unimpaired (CU) control participants.

### 2.2 Exercise testing

All participants completed a treadmill‐based symptom‐limited graded exercise test (GXT) with a clinical exercise physiologist. Heart rate and ECG were assessed at rest and throughout the GXT via 10 electrodes placed on the subject's chest (GE Case, GE Healthcare, Chicago, IL). Participants were fitted with a facemask attached to a metabolic cart (TrueOne 2400, ParvoMedics, Sandy, UT) for the calculation of oxygen consumption and substrate oxidation rates by indirect calorimetry at rest and during each stage of the GXT. The GXT followed a modified Bruce protocol treadmill test (Kaminsky & Whaley, 1998). Participants began at an initial intensity of approximately 2 METs. Intensity was then gradually increased (either by increasing treadmill speed or grade) by approximately 0.3 METs every 20 seconds until volitional fatigue was reached. This protocol was designed so that the final 20 seconds of every 3‐minute stage align with the intensity of the standard Bruce protocol.

### 2.3 Substrate oxidation rate calculations

Absolute rates of FatOx and CHOOx were calculated using the stoichiometric equations derived by Péronnet and Massicotte (Péronnet & Massicotte, 1991), which are:

$$\begin{array}{c} \text{Absolute FatOx}\;\left(\frac{g}{\min}\right)=1.695{\mathrm{VO}}_{2}\left(\frac{L}{\min}\right)\\ \;-1.701{\mathrm{VCO}}_{2}\left(\frac{L}{\min}\right)\text{Absolute CHOOx}\;\left(\frac{g}{\min}\right)\\ \;=4.585{\mathrm{VO}}_{2}\left(\frac{L}{\min}\right)-3.226{\mathrm{VCO}}_{2}\left(\frac{L}{\min}\right) \end{array}$$

Relative oxidation rates were calculated by multiplying the output of these initial equations by 1000, and then dividing the result by each participant's respective body mass in kilograms. Baseline substrate oxidation rate values were calculated as the average of the final minute of standing rest prior to commencement of the GXT. Oxidation rates for each stage were calculated as the average of the final minute of each stage achieved during the GXT. Peak oxidation rates were considered the greatest average rates achieved during a stage of the GXT.

### 2.4 Statistics

All analyses were performed using GraphPad Prism 9. Shapiro–Wilk tests were used to assess normality for each variable. Variables found to be normally distributed were analyzed using unpaired student's *t* tests, whereas variables found to not be normally distributed were analyzed using Mann–Whitney tests. Substrate oxidation rates across rest and the first three stages of the GXT were compared using a two‐way mixed‐effects model ANOVA. This comparison could only be made for these timepoints, as only one aMCI participant was able to reach the fourth stage of the GXT, though several CU participants reached stage 4, and one even reached stage five. The threshold for significance was set at *p* < 0.05. All data are reported as mean ± standard deviation.

## 3 RESULTS

### 3.1 Subject characteristics

All participants were generally healthy and without overt cardiovascular diseases or other chronic illnesses. Participants with aMCI were matched with CU participants based on both age and sex. For two participants in the aMCI group, both systolic and diastolic blood pressure data were not available. For two participants (one in the aMCI group and one in the CU group), resting heart rate data was not available. Additionally, one participant was excluded from the analysis of resting heart rate, as their heart rate was below the expected range. Basic characteristics and resting values are presented in Table 1. All variables were normally distributed, except for BMI, body mass, and resting heart rate. No differences were noted between the aMCI and CU groups for BMI, systolic blood pressure, diastolic blood pressure, or resting heart rate.

**TABLE 1:** Participant characteristics.

	CU	aMCI	*p*‐value
Sex, M/F	6/15	7/15	
Age, years	71 ± 6	74 ± 9	0.314
Height, cm	164 ± 8	163 ± 8	0.795
Body Mass, kg	73 ± 17	71 ± 15	0.678
BMI, kg/m^2^	27 ± 5	26 ± 5	0.957
Resting heart rate, bpm	69 ± 13	66 ± 11	0.634
Systolic blood pressure, mmHg	130 ± 13	134 ± 16	0.377
Diastolic blood pressure, mmHg	78 ± 9	79 ± 10	0.733
Absolute resting FatOx, g/min	0.10 ± 0.03	0.09 ± 0.02	0.126
Relative resting FatOx, mg/kg/min	1.5 ± 0.43	1.4 ± 0.44	0.492
Absolute resting CHOOx, g/min	0.59 ± 0.15	0.51 ± 0.11	0.093
Relative resting CHOOx, mg/kg/min	8.0 ± 2.3	7.5 ± 2.7	0.531
Resting RER	0.77 ± 0.04	0.77 ± 0.05	0.914

*Note*: Data are mean ± SD.

Abbreviations: BMI, body mass index; bpm: beats per minute; RER: respiratory exchange ratio.

### 3.2 Exercise capacity

Results from the GXT are presented in Table 2. For two participants (one in the aMCI group and one in the CU group), time to fatigue data were not available, as the exact start time of their GXTs were not recorded. However, for these participants, data for peak METs, absolute VO_2_ peak, and relative VO_2_ peak were still available. All variables were normally distributed. Time to fatigue was lower in participants with aMCI compared with the CU group (7.2 ± 2.0 vs. 8.7 ± 2.3 min, *p* = 0.033), as was absolute VO_2_ peak (1.3 ± 0.34 vs. 1.6 ± 0.47 L/min, *p* = 0.024). Both peak METs (5.5 ± 1.5 vs. 6.3 ± 1.4, *p* = 0.069) and relative VO_2_ peak (19 ± 5 vs. 22 ± 5 mL/kg/min, *p* = 0.074) tended to be lower in the aMCI group; however, these findings were not statistically significant.

**TABLE 2:** Exercise capacity data.

	CU	aMCI	*p*‐value
Time to fatigue, minutes	8.7 ± 2.3	7.2 ± 2.0	0.033*
Peak METs	6.3 ± 1.4	5.5 ± 1.5	0.069
Absolute VO_2_ peak, L/min	1.6 ± 0.47	1.3 ± 0.34	0.024*
Relative VO_2_ peak, mL/kg/min	22 ± 5	19 ± 5	0.074

*Note*: Data are mean ± SD.

Abbreviation: MET, metabolic equivalent of task.

* *p* < 0.05.

### 3.3 Substrate oxidation

Baseline substrate oxidation rates are reported in Table 1. For two participants (one in the aMCI group and one in the CU group), baseline and peak oxidation rates and respiratory exchange ratio (RER) were not available, as the exact start times of their GXTs were not recorded. Additionally, data from four participants (two in the aMCI group and two in the CU group) were excluded from the analyses of baseline substrate oxidation rates and RER, as their values in at least one of these measures was outside of physiological range. Further, data from two subjects (both in the aMCI group) were excluded from the analysis of peak FatOx, as their values fell outside of physiological range. However, the data for these participants was still included in the analyses of peak CHOOx and peak RER, as these values do not occur at the same point during an exercise test as peak FatOx (Brooks, 1997).

All variables were normally distributed except for resting absolute FatOx. Resting absolute FatOx (0.10 ± 0.03 vs. 0.09 ± 0.02 g/min, *p* = 0.126), resting absolute CHOOx (0.51 ± 0.11 vs. 0.59 ± 0.15 g/min, *p* = 0.093), and RER (0.77 ± 0.05 vs. 0.77 ± 0.04, *p* = 0.914) were not significantly different between groups. Resting relative FatOx (1.5 ± 0.43 vs. 1.4 ± 0.44 mg/kg/min, *p* = 0.492) and resting relative CHOOx (8.0 ± 2.3 vs. 7.5 ± 2.7 mg/kg/min, *p* = 0.126) were also not significantly different between groups.

**FIGURE 1:**
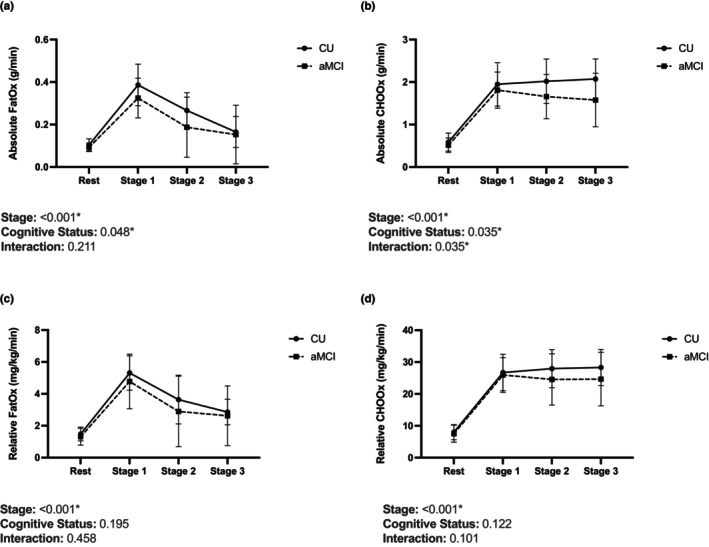
Group data showing (a) absolute FatOx, (b) absolute CHOOx, (c) relative FatOx, and (d) relative CHOOx across rest and the first three stages of the GXT.

Oxidation rates across rest and the first three stages of the GXT are displayed in Figure 1. For absolute FatOx, there were significant main effects of both stage (*p* = <0.001) and cognitive status (*p* = 0.048), though only a significant main effect of stage was present for relative FatOx (*p* = <0.001). For absolute CHOOx, there were significant main effects of both stage (*p* = <0.001) and cognitive status (*p* = 0.035), as well as a significant interaction effect (*p* = 0.035). However, only a significant main effect of stage was present for relative CHOOx (*p* = <0.001).

**FIGURE 2:**
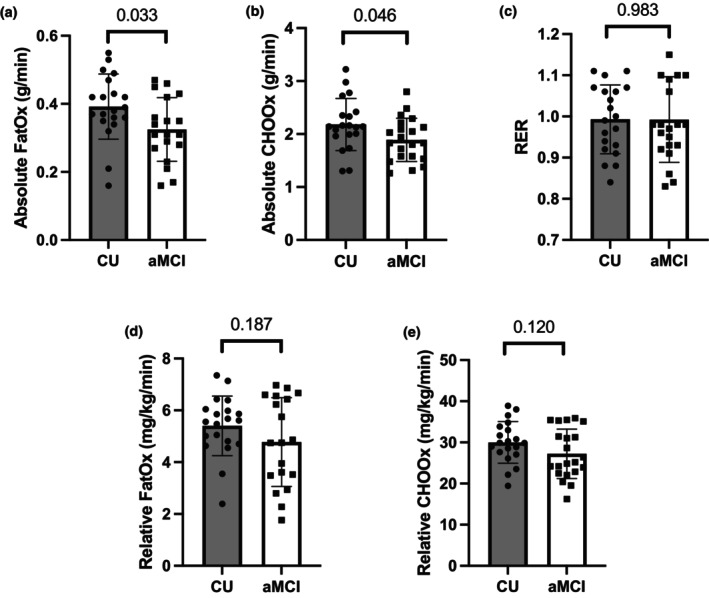
Mean and individual data showing (a) peak rates of absolute FatOx, (b) peak rates of absolute CHOOx, (c) peak RER, (d) peak rates of relative FatOx, and (e) peak rates of relative CHOOx.

Peak substrate oxidation rates are displayed in Figure 2. Peak absolute FatOx (0.33 ± 0.13 vs. 0.39 ± 0.10 g/min, *p* = 0.033) and absolute CHOOx (1.9 ± 0.41 vs. 2.2 ± 0.49 g/min, *p* = 0.046) were significantly lower in aMCI subjects, though no differences in any of peak relative FatOx (5.4 ± 1.1 vs. 4.8 ± 1.7 mg/kg/min, *p* = 0.187), peak relative CHOOx (1.5 ± 0.43 vs. 1.4 ± 0.44 mg/kg/min, *p* = 0.492), or peak RER (0.99 ± 0.10 vs. 0.99 ± 0.08, *p* = 0.983) were observed. Peak FatOx was found to occur at a similar percentage of VO_2_ peak in aMCI and CU subjects (67 ± 10 vs. 63 ± 9%, *p* = 0.468).

## 4 DISCUSSION

The primary finding of this study was that older adults with aMCI have reduced absolute peak FatOx and CHOOx rates compared with age‐ and sex‐matched cognitively unimpaired control participants. We also found reduced absolute oxidation rates across the first three stages of the GXT. Although it is well‐established that resting cerebral metabolism is altered with MCI (De Santi et al., 2001), to our knowledge, no studies have previously explored whole‐body substrate utilization during acute exercise. This is an important consideration, as previous studies have suggested that increases in energy demand are required to elicit detectable differences in metabolic function (Galgani et al., 2008). Thus, substrate oxidation during exercise may be more reflective of metabolic health and flexibility, and therefore a potential biomarker of metabolic dysfunction in MCI.

The exact reasons for reduced substrate oxidation during exercise in aMCI are not known. Recent ex vivo work found impaired skeletal muscle mitochondrial function in MCI (Morris et al., 2021), while other reports indicate altered mitochondrial function in other cell types, such as peripheral blood mononuclear cells and platelets (Mahapatra et al., 2023). Although exercise involves the whole body, the increase in energy expenditure is predominantly driven by skeletal muscle activity. Thus, we speculate that our findings may be primarily driven by reductions in skeletal‐muscle mitochondrial function in aMCI; however, this interpretation requires further validation.

It is important to note that differences in peak substrate oxidation were no longer significantly different following adjustment for body mass. However, mean substrate oxidation rates remained lower in aMCI. There were no group differences in height, body mass, or BMI between the aMCI and CU participants. Thus, it is possible that normalizing body mass may cause overcorrection given the current sample size. Additionally, we did not measure fat free mass in the present study, which is more indicative of metabolically active tissue. Nevertheless, reductions in absolute oxidation rates reflect an overall lower capacity to oxidize substrates during periods of increased energy expenditure and may therefore still reflect important differences in metabolic capacity between the groups.

Our findings may have important implications for metabolic function during submaximal energy expenditures encountered in daily activity. Peak FatOx typically occurs between 45% to 65% of VO_2_ peak (Achten & Jeukendrup, 2004) and is therefore more reflective of substrate utilization at submaximal workloads. Moreover, FatOx was lower in aMCI during the first three stages of graded exercise. Thus, our findings support the idea that metabolic function may be altered in individuals with aMCI during even moderate increases in energy expenditure.

In contrast, CHOOx increases continuously throughout graded exercise, with peak CHOOx occurring near VO_2_ peak (Brooks, 1997). Thus, our finding that absolute peak CHOOx was lower in our aMCI participants may explain our finding that absolute VO_2_ peak was lower in our aMCI group. We also interpret our finding of a significant interaction between cognitive status and stage of exercise as mirroring our aMCI group's lower absolute VO_2_ peak and lesser time to fatigue. The divergent trend of absolute CHOOx across rest and the course of the GXT may reflect an early “leveling‐off” of oxygen consumption in our aMCI group, as might be expected in individuals with a lesser time to fatigue. Therefore, we believe that alterations CHOOx capacity may be less relevant to understanding an individual's overall metabolic capacity in situations outside of explicit exercise.

In the present study, both resting and peak RER were similar between the groups. The lack of differences at rest may be expected despite the potential for underlying differences in skeletal muscle mitochondrial content (Galgani et al., 2008; Holloszy, 2009). However, in contrast to this notion and our present findings, a previous study by Morris et al. found that resting RER was lower in older adults with MCI than in cognitively unimpaired controls (Morris et al., 2021). Their results suggest that older adults with MCI may rely on FatOx to a greater extent at rest than cognitively unimpaired controls, despite the MCI group having reduced lipid‐stimulated skeletal muscle mitochondrial oxidative respiration ex vivo (Morris et al., 2021). We did not observe a difference in resting or peak RER between groups, though peak substrate oxidation rates were reduced in the aMCI group. Considering that RER is expressed as a ratio between the volume of CO_2_ produced and the volume of O_2_ consumed, it cannot account for differences in absolute levels of substrate oxidation when the percentages of substrate contribution are similar. Thus, examining rates of substrate oxidation during exercise, when energy demand is significantly elevated, may unmask metabolic differences between older adults with MCI and cognitively unimpaired control participants that are not apparent at rest. We also found that older adults with aMCI had significantly lower absolute VO_2_ peaks compared with controls. The aMCI group tended to have lower relative VO_2_ peaks as well, although these results were not statistically significant. Our finding that relative VO_2_ peak did not differ between older adults with MCI and cognitively unimpaired controls is in agreement with the findings of Morris et al. (Morris et al., 2021). However, in contrast to their finding that time to fatigue was only reduced in a subset of MCI patients (Morris et al., 2021), we found that time to fatigue was significantly reduced in the aMCI group.

These findings regarding exercise capacity may add additional context to our substrate oxidation data, as some have speculated that alterations in systemic metabolism may be a greater reflection of differences in VO_2_ peak rather than mitochondrial function (Dela & Helge, 2013; Holloszy, 2009). However, the present study found that absolute peak substrate oxidation rates were lower in older adults with aMCI despite no significant differences in relative VO_2_ peak being observed. This potentially indicates that mitochondrial function may be altered prior to, or independently from, VO_2_ peak in individuals with aMCI. In support of this interpretation, recent preclinical research found that mitochondrial adaptations were altered in response to exercise training in mice expressing an AD‐like phenotype (Brisendine et al., 2024). Importantly, the AD‐like mice in the study still experienced increases in exercise capacity despite their altered mitochondrial adaptive responses (Brisendine et al., 2024), indicating that AD pathology may affect the mitochondria separately from VO_2_ peak.

Based on the findings of the current study, as well as the others previously discussed, we suggest there may be an additional means by which alterations in metabolic function may affect those with cognitive impairment during everyday activities. A reduced ability to upregulate FatOx at submaximal intensities may compromise the brain's access to circulating glucose during periods of increased energy expenditure, such as those that occur during activities of daily living. The brain has a limited capacity for energy storage and predominantly relies on circulating glucose for energy production (Magistretti & Allaman, 2022). Increased FatOx capacity in peripheral tissues may therefore spare circulating glucose for use by the brain during periods of increased metabolic demand. However, reduced metabolic flexibility, including the ability to efficiently oxidize lipids in the periphery, may strain the supply of energy to the brain in conditions such as MCI, where it is already known to be compromised (De Santi et al., 2001). The present study suggests that individuals with aMCI have similar levels of resting substrate oxidation but a reduced absolute ability to upregulate FatOx, making them potentially vulnerable to increases in cerebral metabolic demand, like those that occur during everyday activities. Thus, interventions aimed at enhancing whole body metabolic flexibility may be particularly beneficial for older adults with aMCI.

## 5 LIMITATIONS

There are some limitations to the current study. First, no direct measure of mitochondrial function was performed. While it is likely that reductions in mitochondrial oxidative capacity may underlie the present findings, this cannot be conclusively determined given the current data. Second, no blood‐based measures of systemic metabolic function were collected as a part of the parent study, meaning that we cannot determine whether our results occurred in the presence of larger disruptions in metabolic function, such as having individuals with metabolic syndrome in our aMCI group. Third, no measures of habitual physical activity (either directly measured or self‐reported) were recorded as part of the parent study, making it difficult to fully understand the context behind the differences in absolute substrate oxidation rates, as it is possible that the CU group was more habitually active. Fourth, no measure of specifically lean body mass was available for the present study, which may have limited our ability to optimally normalize oxidation rates. Finally, there was no standardized meal given to the subjects prior to their participation in the study. It is possible that differences in meal consumption on the night before or morning prior to participation in the GXT may have influenced the rates of FatOx and CHOOx observed, as previous work has demonstrated differences in pre‐exercise meal content influence substrate oxidation rates (Whitley et al., 1998). These limitations should each be addressed by future studies investigating mitochondrial function in aMCI and its potential role in altering systemic metabolism and cognitive function.

## 6 CONCLUSION

In conclusion, our findings suggest that older adults with aMCI have reduced rates of absolute substrate oxidation compared to age‐ and sex‐matched cognitively unimpaired control participants. Future studies should examine whether changes in mitochondrial function underlie such changes, determine whether these may precede larger changes in metabolic function, and identify to what extent impaired skeletal muscle FatOx may affect brain energy supply and cognitive function.

## AUTHOR CONTRIBUTIONS

J.M.E., A.M.L., M.L.C., D.G.E., M.L.C., C.L.J., and C.R.M. conceived and designed the research; M.L.O. performed experiments; N.A.R. and T.M.D. analyzed data; N.A.R., M.K.K., T.M.D., J.M.E., A.M.L., M.L.C., D.G.E., M.L.C., C.L.J., and C.R.M. interpreted results of the experiments; N.A.R. prepared tables and figures; N.A.R. drafted the manuscript; All authors reviewed and edited the initial draft and approved the final version of the manuscript.

## FUNDING INFORMATION

Funding for this study was provided by NIH grants R01 AG058853 (Johnson), K01 AG054731 (Martens), and P20GM113125 (Edwards), and a grant from the Howard W. Swank, Alma K. Swank, and Richard Kemper Swank Foundation (Martens). Additional resources were provided by the Delaware Center for Cognitive Aging Research and the UD Center for Human Research Coordination.

## CONFLICTS OF INTEREST STATEMENT

The authors have no conflicts of interest, financial or otherwise, to report.

## Data Availability

Data are available from the corresponding author upon reasonable request.
